# Advancements in Herpes Zoster Diagnosis, Treatment, and Management: Systematic Review of Artificial Intelligence Applications

**DOI:** 10.2196/71970

**Published:** 2025-06-30

**Authors:** Dasheng Wu, Na Liu, Rui Ma, Peilong Wu

**Affiliations:** 1 Department of Pain Management, Jilin Provincial People's Hospital, No. 1183, Gongnong Road, Chaoyang District, Changchun, 130021, China, 86 0431-85595114

**Keywords:** herpes zoster, artificial intelligence, deep learning, machine learning, dermatology

## Abstract

**Background:**

The application of artificial intelligence (AI) in medicine has garnered significant attention in recent years, offering new possibilities for improving patient care across various domains. For herpes zoster, a viral infection caused by the reactivation of the varicella-zoster virus, AI technologies have shown remarkable potential in enhancing disease diagnosis, treatment, and management.

**Objective:**

This study aims to investigate the current research status in the use of AI for herpes zoster, offering a comprehensive synthesis of existing advancements.

**Methods:**

A systematic literature review was conducted following PRISMA (Preferred Reporting Items for Systematic Reviews and Meta-Analyses) guidelines. Three databases of Web of Science Core Collection, PubMed, and IEEE were searched to identify relevant studies on AI applications in herpes zoster research on November 17, 2023. Inclusion criteria were as follows: (1) research articles, (2) published in English, (3) involving actual AI applications, and (4) focusing on herpes zoster. Exclusion criteria comprised nonresearch articles, non-English papers, and studies only mentioning AI without application. Two independent clinicians screened the studies, with a third senior clinician resolving disagreements. In total, 26 articles were included. Data were extracted on AI task types; algorithms; data sources; data types; and clinical applications in diagnosis, treatment, and management.

**Results:**

Trend analysis revealed an increasing annual interest in AI applications for herpes zoster. Hospital-derived data were the primary source (15/26, 57.7%), followed by public databases (6/26, 23.1%) and internet data (5/26, 19.2%). Medical images (9/26, 34.6%) and electronic medical records (7/26, 26.9%) were the most commonly used data types. Classification tasks (85.2%) dominated AI applications, with neural networks, particularly multilayer perceptron and convolutional neural networks being the most frequently used algorithms. AI applications were analyzed across three domains: (1) diagnosis, where mobile deep neural networks, convolutional neural network ensemble models, and mixed-scale attention-based models have improved diagnostic accuracy and efficiency; (2) treatment, where machine learning models, such as deep autoencoders combined with functional magnetic resonance imaging, electroencephalography, and clinical data, have enhanced treatment outcome predictions; and (3) management, where AI has facilitated case identification, epidemiological research, health care burden assessment, and risk factor exploration for postherpetic neuralgia and other complications.

**Conclusions:**

Overall, this study provides a comprehensive overview of AI applications in herpes zoster from clinical, data, and algorithmic perspectives, offering valuable insights for future research in this rapidly evolving field. AI has significantly advanced herpes zoster research by enhancing diagnostic accuracy, predicting treatment outcomes, and optimizing disease management. However, several limitations exist, including potential omissions from excluding databases like Embase and Scopus, language bias due to the inclusion of only English publications, and the risk of subjective bias in study selection. Broader studies and continuous updates are needed to fully capture the scope of AI applications in herpes zoster in the future.

## Introduction

In recent years, the application of artificial intelligence (AI) technology in the field of medicine has garnered widespread attention [1-4]. With continuous technological advancements and breakthroughs, AI has begun to play an increasingly significant role in disease research and clinical practice [5,6]. Herpes zoster, caused by the varicella-zoster virus (VZV), is a disease characterized by a rash and pain in a dermatomal distribution, significantly impacting patients’ quality of life [7]. The rising incidence of herpes zoster underscores the crucial need for further research and understanding of this condition.

The prevalence of herpes zoster and the risk of complications are on the rise due to the aging population [8,9]. Despite recent advancements, the prevention, diagnosis, treatment, and management of herpes zoster remain challenging. Early detection and accurate diagnosis of herpes zoster are critical for preventing complications such as postherpetic neuralgia (PHN). Additionally, the prediction of treatment outcomes and the management of long-term complications remain areas requiring further improvement.

The emergence of AI technology offers promising solutions to these challenges. AI technologies can process large volumes of medical data and support clinical decision-making, which brought new opportunities and prospects for herpes zoster research [10,11]. The use of AI in herpes zoster research holds great potential for expediting disease diagnosis, improving treatment efficacy, and offering novel perspectives to researchers. Specifically, AI holds tremendous potential in facilitating early detection of herpes zoster by leveraging its capacity to analyze extensive clinical data [12]. Through this analysis, AI algorithms can identify distinctive attributes associated with herpes zoster, empowering physicians to make accurate and timely diagnoses [13]. Furthermore, the integration of AI with image recognition technology enables rapid identification of herpes zoster by analyzing visual representations of skin lesions [14]. This capability empowers health care professionals to promptly administer appropriate treatments to affected individuals.

However, there is currently no comprehensive synthesis of AI applications specifically in herpes zoster research, making it difficult to identify research trends, understand prevalent methodologies, and uncover potential areas for further exploration. This gap underscores the need for a systematic review that consolidates existing knowledge, identifies key advancements, and highlights future research directions in this rapidly evolving field.

**Figure 1.**
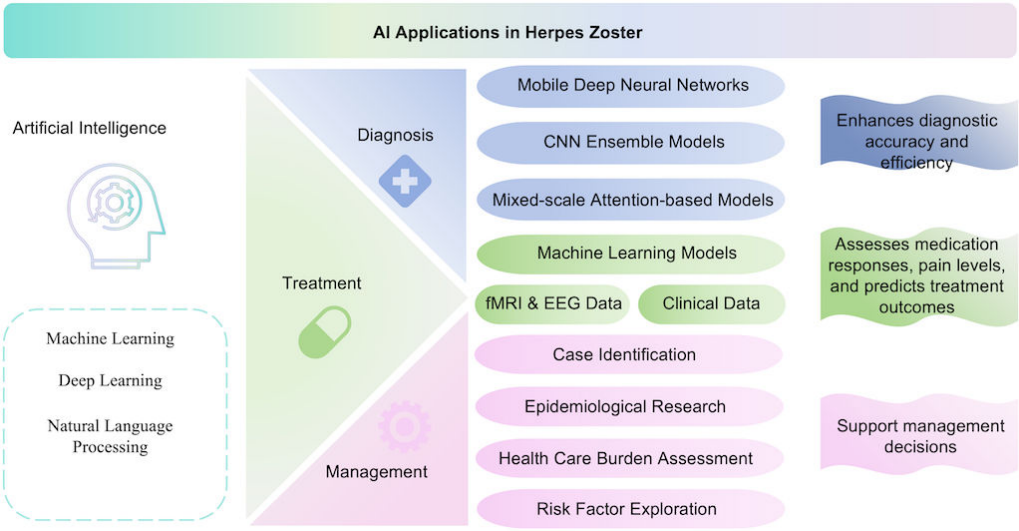
Overview of artificial intelligence applications in herpes zoster. CNN: convolutional neural network; EEG: electroencephalogram; fMRI: functional magnetic resonance imaging.

To address this gap, this study aims to provide a comprehensive and systematic overview of the current landscape of AI applications in herpes zoster research. We hypothesize that AI has significantly advanced herpes zoster research by enhancing diagnostic accuracy, predicting treatment outcomes, and optimizing disease management. By synthesizing these advancements, this study aims to offer valuable insights into how AI is transforming herpes zoster research and to lay the groundwork for future innovations in the field (Figure 1). Specifically, we seek to (1) identify the predominant AI algorithms, tasks, and data sources used in herpes zoster research; (2) categorize AI applications into diagnosis, treatment, and management to offer a structured understanding of AI’s impact across these domains; and (3) highlight existing challenges and propose directions for future research.

## Methods

### Data Collection and Search Strategies

This systematic review was prospectively registered on the Open Science Framework (registration number 5xj7w). We followed the PRISMA (Preferred Reporting Items for Systematic Reviews and Meta-Analyses) 2020 framework to screen and analyze the literature (Checklist 1) [15].

We selected 3 major databases, PubMed (biomedical and health sciences), Web of Science Core Collection (multidisciplinary science), and IEEE Xplore (computer science and engineering), to ensure cross-disciplinary coverage of the intersection between AI and herpes zoster research. These databases were chosen for their indexing quality and relevance to the scope of our review. The search keywords used in our search strategy were selected based on a combination of clinical expert suggestions and previous literature reviews [16]. The search query and keywords are as follows: #1: TS = (“herpes zoster” OR “shingles” OR “zoster” OR “postherpetic neuralgia”), #2: TS = (“artificial intelligence” OR “machine learning” OR “deep learning” OR “natural language processing” OR “data mining” OR “image analysis” OR “pattern recognition” OR “computer vision”), and final dataset: #1 AND #2. The complete search strategies for all databases, including exact search strings and search results have been provided in Multimedia Appendix 1 [10-14,17-37]. A total of 161 related documents were retrieved. No time restrictions were applied during the database search to ensure no relevant studies were missed, given the relatively limited research in this field and the rise of AI applications, particularly deep learning, in clinical domains over the past decade.

### Inclusion and Exclusion Criteria

In order to ensure the accuracy of the screening results, we designed inclusion and exclusion criteria and established an expert panel that consisted of 3 domain researchers. Two of the researchers came to screen the literature and discrepancies in results were discussed and arbitrated by another researcher.

The inclusion criteria comprised of the following: (1) the study should be research articles; (2) the study should be published in the English language; (3) the study should actually apply AI technologies; and (4) the study must apply AI directly to the clinical diagnosis, treatment, or management of herpes zoster. The exclusion criteria comprised of the following: (1) the research types are reviews, letters, comments, books, and preprint; (2) the language is not English; (3) AI technologies are not used or only mentioned in background part without practical application; and (4) the study focuses on areas outside of clinical applications, such as health economics or public health prevention strategies for herpes zoster.

### Quality Appraisal

To assess the methodological quality and risk of bias of the included studies, we used the Joanna Briggs Institute (JBI) Critical Appraisal Checklist for Analytical Cross-Sectional Studies [38], which is a validated tool suitable for evaluating observational studies. This checklist covers 8 domains including study population, measurement reliability, identification and control of confounding factors, and statistical analysis, which together provide a structured evaluation of both methodological rigor and potential risk of bias. Two independent researchers conducted the quality appraisal process. Any disagreements were resolved through discussion with a senior physician experienced in evidence-based medicine. Each study was scored based on the JBI checklist, which consists of 8 items evaluating various methodological domains. The total score for each study was used to categorize its quality as follows: a score ≥6 was considered high quality, a score ≥4 and<6 was considered medium quality, and a score <4 was considered low quality.

### Data Process

We manually extracted the following basic data from the included articles: title, country, institution, author, journal, publication date, etc. Additionally, we read the full text and extracted detailed information, including the data source, data type, AI task type, and AI technology. A deductive coding approach was used, guided by predefined categories aligned with the research objectives. To ensure the accuracy of extracted information, 2 clinicians (NL and RM) independently extracted all information. If there were any disagreements, a third senior clinician (DW) discussed with them until a consensus was achieved.

### Data Analysis

First, we used content analysis to gain a comprehensive understanding of the landscape of AI technologies in herpes zoster research, including diagnosis, treatment, and health management. Second, we analyze the application of AI technology in herpes zoster research from 4 aspects: AI task types, AI algorithms, data sources, and data types.

### Ethical Considerations

The Research Ethics Committee of Jilin Provincial People’s Hospital has determined that this review does not involve any human or patient data and is therefore exempt from ethical review.

## Results

### Quality Appraisal of Included Studies

**Figure 2.**
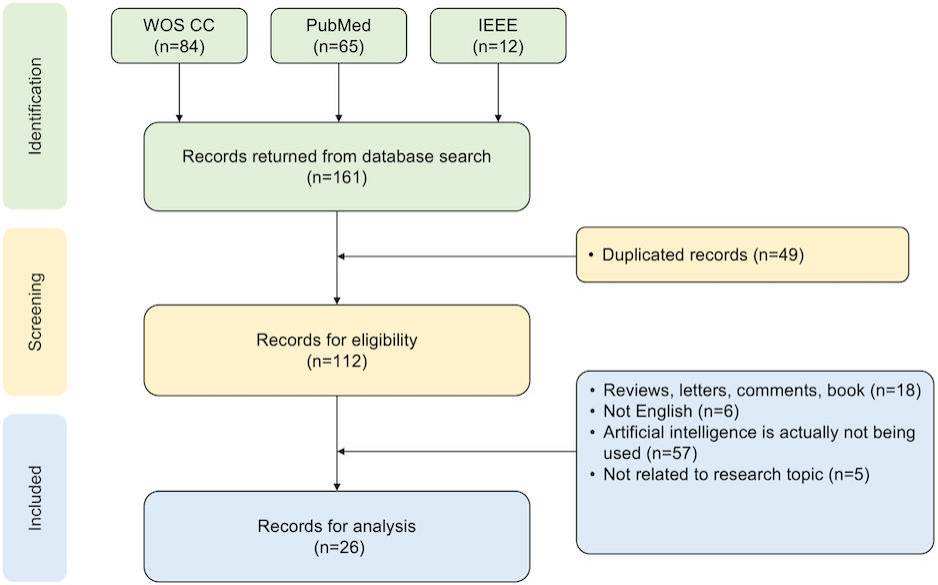
Publications search flow diagram. WOS CC: Web of Science Core Collection.

The flowchart of publication screening is shown in Figure 1. A total of 161 articles were retrieved in this study, of which 84 articles were from the Web of Science database, 65 articles were from the PubMed database, 12 articles were from the IEEE database, and 49 articles remained after deduplication. In order to ensure the accuracy of the screening results, we designed inclusion and exclusion criteria and formed an expert panel consisting of 3 clinicians from the Pain Department of Jilin Provincial People’s Hospital, all specializing in chronic pain management and have experience in diagnosing and treating herpes zoster. Two clinicians (NL and RM) screened all the articles, and the third senior clinician reviewed and resolved any disagreements. Finally, 26 articles were included in the analysis for this study (Figure 2 and Multimedia Appendix 1 [10-14,17-37]).

The quality appraisal of the included studies is summarized in Table 1, which presents the study characteristics along with their overall quality ratings. Out of the 26 studies included in this review, 21 (81%) studies were rated as high quality, 5 (19%) studies were rated as moderate quality, and 1 (4%) study was rated as low quality based on the JBI criteria. This suggests that the majority of studies applying AI to herpes zoster diagnosis and management were methodologically sound. The full results of the JBI appraisal, including item-by-item scoring for each study, are detailed in Multimedia Appendix 2 [10-14,17-37].

**Table 1.** Key characteristics of the included studies, including publication year, data sources and types, artificial intelligence (AI) tasks, algorithms used, and the overall quality appraisal based on the Joanna Briggs Institute critical appraisal tool.

Study	Year	Data source	Data type	AI task	AI algorithms	Overall appraisal
Eze et al [19]	2023	Internet	Medical image	Classification	NN^a^	Medium
Wang et al [35]	2023	Hospital	Structured data	Classification	LR^b^; RF^c^; DT^d^; NN; SVM^e^; k-NN^f^; XGBoost^g^	High
Kovalishyn et al [32]	2023	Public database	Structured data	Regression	NN	Medium
Zhou et al [23]	2022	Hospital	Omics data	Classification	LR; RF; SVM	High
Mejia et al [33]	2022	Public database	Medical image	Classification	NN	Low
Yu et al [14]	2022	Hospital	Medical image	Segmentation	NN	High
Wei et al [20]	2022	Hospital	Electroencephalogram data	Classification	k-NN	Medium
Lanera et al [10]	2022	Public database	Electronic medical record	Classification	NN	High
Ho et al [31]	2022	Hospital	Electronic medical record	Classification	NLP^h^; k-NN	High
Zheng et al [11]	2022	Hospital	Medical image	Classification	SVM	High
Nayak et al [34]	2022	Public database	Medical image	Classification; Segmentation	k-NN	High
Zheng et al [37]	2021	Hospital	Electronic medical record	Classification	NLP	High
Back et al [17]	2021	Public database	Medical image	Classification	NN	Medium
Zhou et al [21]	2021	Hospital	Structured data	Classification	LR; RF; SVM; NB^i^	High
Mathur et al [18]	2021	Public database	Medical image	Classification	NN	High
Wang et al [12]	2020	Hospital	Structured data	Classification	LR; RF	High
Chen et al [27]	2020	Hospital	Electronic medical record	Classification	XGBoost	High
Li et al [13]	2020	Hospital	Structured data	Classification	Other	High
Burlina et al [25]	2020	Internet	Medical image	Classification	NN	High
Zheng et al [36]	2019	Hospital	Electronic medical record	Classification	NLP	High
Burlina et al [26]	2019	Internet	Medical image	Classification	NN	High
Turner et al [22]	2018	Hospital	Electronic medical record	Classification	NLP	High
Gilbert et al [30]	2018	Hospital	Structured data	Regression	Other	High
Curtis et al [28]	2017	Internet	Web-based text	Classification	NLP	High
Gianfrancesco et al [29]	2017	Hospital	Electronic medical record	Classification	LR; Other; RF; SVM; Other; NB	High
Al-Jefri et al [24]	2017	Internet	Web-based text	Classification	NB; k-NN; SVM; LR; NN	High

a NN: neural network.

b LR: logistic regression.

c RF: random forest.

d DT: decision tree.

e SVM: support vector machine.

f k-NN: k-nearest neighbor.

g XGBoost: Extreme Gradient Boosting.

h NLP: natural language processing.

i NB: Naive Bayes.

### Fundamentals of AI Technology

**Figure 3.**
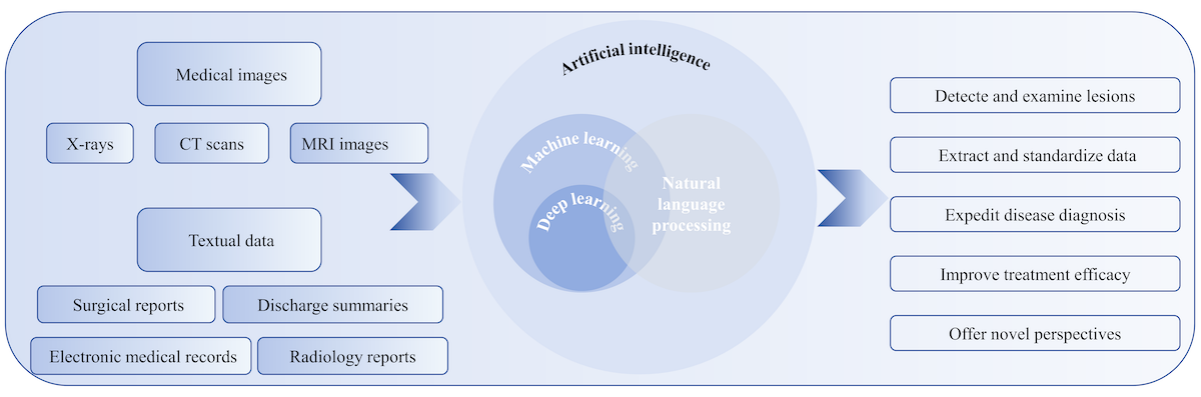
Types of AI and their application in the medical field. CT: Computed Tomography; MRI: magnetic resonance imaging.

As shown in Figure 3, AI technology encompasses various components, including machine learning (ML), deep learning, and natural language processing (NLP) [39-43]. The progressive informatization of hospitals has resulted in the accumulation of extensive image and text data [44]. This data comprises various types of medical images such as x-rays, computed tomography scans, and magnetic resonance imaging images. Leveraging AI techniques such as convolutional neural networks (CNNs), these medical images can be accurately analyzed and identified, assisting doctors in detecting and examining lesions and abnormalities [45]. In addition to image data, hospitals also possess a wealth of textual data, including surgical reports, discharge summaries, radiology reports, and so on [46-48]. NLP technology plays a crucial role in efficiently processing this textual information. By using NLP techniques, clinicians and researchers can extract and standardize data, enabling them to navigate through large volumes of electronic medical records (EMRs) more effectively [49,50]. Furthermore, NLP facilitates the identification of concealed associations and patterns within the data, thus empowering in-depth medical research and enhancing clinical decision support.

### AI Applications in Herpes Zoster Diagnosis

To date, different AI-aided diagnosis models have been developed. Three of these articles specifically focus on supporting the diagnosis of herpes zoster, using ML algorithms on clinical image datasets for herpes zoster detection. Back et al [17] proposed a robust mobile deep neural network (DNN) approach for the early diagnosis of herpes zoster, using AI to address visual distortions in clinical images captured by mobile devices. Through knowledge distillation from ensemble via curriculum training, a robust DNN model was trained with low computational cost. The knowledge distillation from ensemble via curriculum training model significantly improved robustness against image corruptions and achieved a high accuracy of 93.5% and a low mean corruption error of 67.6%, outperforming DNN ensembles and making it suitable for mobile skin lesion analysis for herpes zoster diagnosis [17]. Mathur et al [18] introduced a novel CNN ensemble model to detect COVID-19–related skin lesions from clinical images. In COVID-19 cases with a herpes zoster infection, the model achieved a sensitivity of approximately 86.4%, specificity of approximately 99.4%, positive predictive value of approximately 85.2%, negative predictive value of approximately 99.4%, and area under the curve of approximately 0.97. This was an ML study focused on the automated detection of skin images. The model exhibited resilience in identifying skin lesions across diverse skin tones, despite the limitation of having limited data from darker skin tones [18]. Eze et al [19] introduced a mixed-scale dense convolution, self-attention, hierarchical feature fusion, and attention-based contextual information (mixed-scale hierarchical attention [MSHA]) model for skin lesion detection in chickenpox and shingles cases. The MSHA model achieved an accuracy of 95.0% and a loss of 0.104%, outperforming state-of-the-art models. The MSHA model served as a reliable tool for accurate and timely diagnosis of dermatological conditions [19].

AI has made remarkable progress in the field of medical diagnosis, especially in the identification of herpes zoster. This includes advancements such as robust mobile DNNs, CNN ensemble models, and mixed-scale attention-based models. These AI models exhibit promising potential in enhancing the accuracy and efficiency of herpes zoster diagnosis.

### AI Applications in Herpes Zoster Treatment

Herpes zoster spreads across the skin, causing pain and blistering [51]. The treatment of herpes zoster relies on medication; yet, the ability to predict medication responses or pain levels for patients remains a significant challenge. In this context, the application of AI in herpes zoster treatment holds immense promise. Zheng et al [11] used a deep autoencoder algorithm coupled with resting-state functional magnetic resonance imaging (fMRI) technology to evaluate the clinical efficacy of pregabalin in treating PHN. Among 40 patients with PHN randomly divided into treatment and control groups, the algorithm predicted whether patients’ symptoms had improved. This algorithm offered valuable insights for treatment strategies [11]. Wei et al [20] established an ML model for herpes zoster treatment prediction of medication responses from electroencephalogram (EEG) data. The study compared the classification performances of 4 prediction models: linear discriminant analysis, k-nearest neighbors (k-NNs), support vector machine (SVM), and random forest (RF) in 70 patients with herpes zoster with different drug treatment outcomes. The results indicate that the k-NNs model achieved the best performance, with an accuracy of 80%, sensitivity of 82.5%, specificity of 77.7%, and an area under the curve of 0.85 [20]. Zhou et al [21] fitted 4 ML models: logistic regression, RF, SVM, and Naïve Bayes. These models were trained using clinical characteristics obtained during patient admission to accurately identify medication-resistant pain (MRP). Notably, the predictive accuracy of this integrated approach stands at 0.917, surpassing the performance of previous imaging or EEG-based methodologies [21].

AI techniques have emerged as promising tools in evaluating herpes zoster treatment. Through AI-driven research that leverages fMRI, EEG, and clinical characteristics, medication responses, and pain levels have been effectively assessed. ML models, such as deep autoencoders, have proven adept at predicting medication responses and identifying MRP, thus paving the way for better patient care.

### AI Applications in Herpes Zoster Management

In recent years, with the rapid development of big data and AI technologies, NLP and ML have increasingly demonstrated their immense potential in the medical field, particularly in the management and diagnosis of herpes zoster (including its ocular complication, herpes zoster ophthalmicus). A series of studies have explored the use of EMRs and NLP techniques to identify, predict, and analyze cases of varicella zoster. Lanera et al [10] developed a deep learning approach using a recurrent neural network (NN) with bidirectional gated recurrent units to estimate the incidence of VZV infection from routinely collected ambulatory records in Pedianet, an Italian pediatric primary care database. The model, trained on free text data from 2004 to 2014 in the Veneto region, achieved a maximum area under the receiver operating characteristic curve of 95.30% over time, demonstrating high accuracy in identifying VZV infection cases. The absolute bias in estimates of VZV infection remained below 1.5% in the final 5 years analyzed, indicating the potential for large-scale use of EHRs in clinical outcome predictive modeling and the establishment of high-performing systems in other medical domains [10]. Turner et al [22] used an NLP algorithm to analyze 22 million EMR transactions from New Zealand general practices between 2005 and 2015, quantifying the incidence of primary care presentations for herpes zoster. The results indicated that herpes zoster consultations were infrequent in primary care [22]. Li et al [13] built a prediction model using TREENET algorithms. They identified 62 variables associated with PHN in patients with herpes zoster at the Sichuan Hospital of Traditional Chinese Medicine. They selected 1303 cases and 2958 indicators to build a predictive model, which achieved high performance with receiver operating characteristic curve values of 0.985 (training) and 0.752 (testing), and 70.27% accuracy. The key risk factors for PHN that they identified included age, mean erythrocyte hemoglobin concentration, sodium, and serum uric acid. Their findings laid a solid foundation for further exploring the comprehensive management and prevention of PHN [13].

These studies have not only improved the accuracy and efficiency of case identification but also provided powerful support for population-based epidemiological research and public health decision-making. Through deep learning algorithms and ML models, researchers have been able to quantify the incidence of primary care presentations for herpes zoster, assess herpes zoster-associated health care burden, and explore risk factors related to PHN and other complications. These efforts have offered new perspectives and tools for the comprehensive management and prevention of herpes zoster.

### Analysis of Data Resources and AI Technologies in Herpes Zoster

**Figure 4.**
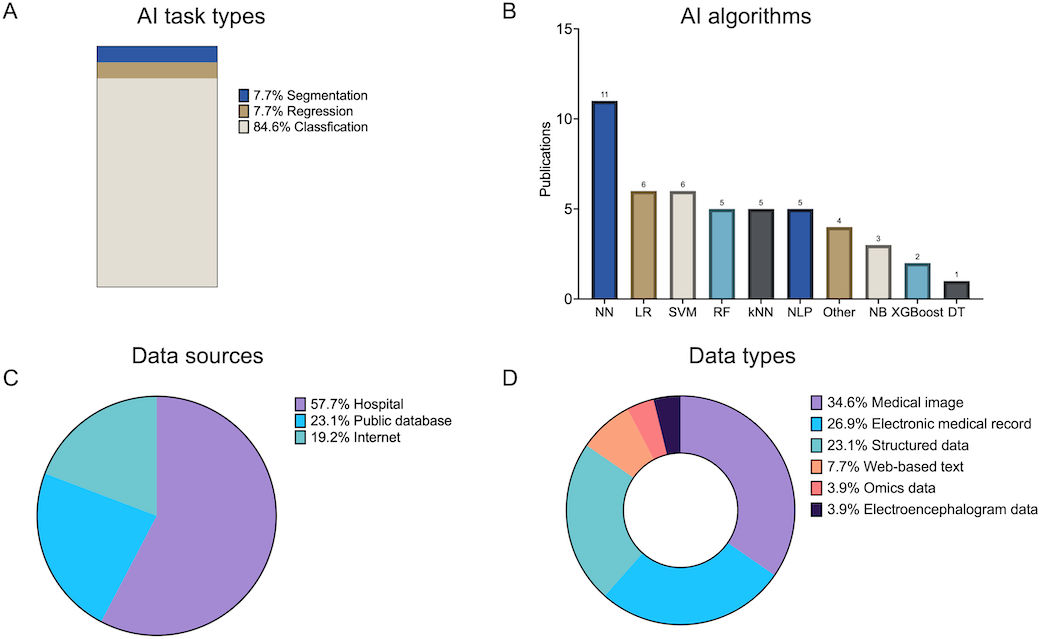
Distributions across AI tasks, algorithms, and data. (**A**) Distribution of AI task types. (**B**) Distribution of algorithms. (**C**) Distribution of data sources. (**D**) Distribution of data types. AI: artificial intelligence; DT: decision tree; k-NN: k-nearest neighbor; LR: logistic regression; NB: Naive bayes; NLP: natural language processing; NN: neural network; RF: random forest; SVM: support vector machines; XGBoost: Extreme Gradient Boosting.

We analyze the application of AI technology in herpes zoster research from 2 aspects: AI task types and AI algorithms (Figures 4A and B).

For AI tasks (Figure 4A), the relevant studies primarily focus on classification tasks (22/26, 84.6%). These classification tasks involve categorizing textual and image data, including both binary and multiclass classification tasks. Additionally, regression tasks (2/26, 7.7%) and segmentation tasks (2/26, 7.7%) are also considered.

For AI algorithms (Figure 4B), the most commonly used one is NN algorithms (11/26, 42.3%). Classical ML algorithms are the next most used, including logistic regression (6/26, 23.1%), SVMs (6/26, 23.1%), RFs (5/26, 19.2%), k-NN (5/26, 19.2%), Naive Bayes (3/26, 11.4%), Extreme Gradient Boosting (2/26, 7.7%), and decision trees (1/26, 3.9%). When dealing with text data, NLP algorithms are commonly used for feature representation and analysis (5/26, 19.2%), followed by classification tasks using ML algorithms. The NLP algorithms encompass various methodologies, such as rule-based and dictionary-based approaches, as well as some publicly available NLP platforms. Conversely, for image data, NN algorithms are often used as the primary technique for processing image data, enabling a comprehensive exploration of relevant patterns.

Then, we conducted a comprehensive analysis of various aspects encompassing data sources and data types. As shown in Figure 4C, for data sources, the analysis revealed that a majority of the studies relied on data collected from hospitals (15/26, 57.7%) as their primary source, followed by public databases (6/26, 23.1%) and the internet (5/26, 19.2%). The internet served as a diverse and expansive platform, offering various sources of data. For instance, researchers accessed image data through popular search engines such as Google and Bing, while social textual data from platforms like Twitter. For data types, the most commonly used type was a medical image (9/26, 34.6%), followed by EMRs (7/26, 26.9%), structured data (6/26, 23.1%), web-based text (2/26, 7.7%), omics data (1/26, 3.9%), and electrocardiogram data (1/26, 3.9%; Figure 4D).

## Discussion

### Principal Findings

The application of AI technology in herpes zoster research holds great promise for advancing disease diagnosis, treatment, and prevention [10,12,23]. Our study revealed the diversity and complexity of data sources in the field of AI research for herpes zoster. First, the hospital was the primary source of data, emphasizing the importance of real-world clinical data in driving AI advancements. Hospitals internally maintain vast records including medical histories, patient information, medical reports, and laboratory results. These data can be used to investigate aspects such as disease prevalence, treatment effectiveness, and patient survival rates. Access to hospital data typically requires patient consent and compliance with privacy regulations. Second, data can be obtained from public databases. These databases are established to facilitate medical research and public health initiatives. Examples include the National Institutes of Health in the United States, which maintains a range of public databases such as genomic databases, clinical trial databases, health statistics databases, and literature databases like PubMed [52]. These databases provide a wealth of health care information and the latest scientific research findings. Researchers can access these public databases to conduct various health care studies. Additionally, the internet serves as a unique data source. It is a crucial avenue for accessing health care information. People can acquire various medical knowledge, case reports, physician advice, and patient experiences through search engines, medical websites, health forums, and similar platforms. Such data can be used to investigate aspects such as disease etiology, symptoms, and treatment methods.

The use of various data types, including medical images, textual data, omics data, and electrocardiogram data, showcases the multidimensional approach of AI in capturing and analyzing different aspects of the disease. This comprehensive data analysis enables AI algorithms to perform classification, regression, and segmentation tasks, with a strong focus on classification. The classification tasks were the most commonly studied in the context of herpes zoster. This indicates the focus on categorizing and identifying patterns in textual and image data to aid in accurate diagnosis and treatment decisions. NN algorithms, particularly CNNs, are widely used for image analysis, while classical ML algorithms and NLP techniques are applied to textual data analysis. This is consistent with the ability of deep learning-based algorithms to effectively analyze and process complex image and textual data. Moreover, NLP techniques enable efficient processing of textual information, facilitating data extraction, standardization, and analysis of large volumes of EMRs in this field.

The integration of AI into clinical practice offers several benefits. AI-powered diagnostic models, such as CNNs, can assist clinicians in identifying herpes zoster lesions more accurately and swiftly, reducing the risk of misdiagnosis, especially in atypical cases [17-19]. These models can also be deployed on mobile devices, enabling real-time assessment and enhancing accessibility for health care providers in remote areas [17]. Additionally, AI-driven models using fMRI, EEG, and clinical data can help predict medication responses, optimize drug selection, and identify patients at risk of MRP, enabling more personalized and effective herpes zoster treatment [11,20,21]. Incorporating these AI tools into clinical workflows could optimize treatment decisions and improve patient outcomes. Furthermore, AI algorithms analyzing electronic health records facilitate early detection of complications, such as PHN, allowing for timely interventions [13]. Additionally, by streamlining data processing and analysis, AI reduces the administrative burden on health care providers, enabling them to focus more on patient care [10,22]. Overall, the findings of this study demonstrate the potential of AI in advancing herpes zoster research.

Our quality appraisal of the included studies using the JBI Critical Appraisal Checklist indicates that the overall methodological rigor in this field is relatively high, with over 80% of studies scoring in the high-quality range. This strengthens the reliability of our synthesized findings and suggests that the application of AI in herpes zoster research is being pursued with considerable methodological care. However, the presence of a small number of moderate and low-quality studies highlights the need for continued attention to study design, particularly regarding confounding factors and outcome measurement. Future research should aim to address these aspects more explicitly to further improve the robustness and reproducibility of AI applications in this domain.

### Future Directions for AI-Based Herpes Zoster Research

There are several areas that need further exploration and development. First, collaboration between countries and institutions should be encouraged to foster knowledge sharing and promote international research efforts. Collaborative initiatives can facilitate the exchange of expertise, resources, and data, leading to more comprehensive and impactful research outcomes. Second, the lack of standardized datasets and protocols hinders the comparison and reproducibility of studies. Collaborative efforts are needed to establish standardized guidelines and datasets to ensure the validity and reliability of AI applications in herpes zoster research. Third, while classification tasks dominate the current research landscape, there is a need to explore other AI tasks such as regression and segmentation. Regression models can provide valuable insights into disease progression, treatment response, and prognosis, while segmentation algorithms can enhance the analysis of complex image data and assist in precise lesion localization. Finally, the advancement of AI technology should be coupled with efforts to ensure data quality, privacy, and ethical considerations. As AI relies on extensive data, maintaining data integrity, privacy protection, and adherence to ethical guidelines are critical to building trust and ensuring the responsible use of AI in herpes zoster research.

### Limitations

This study has several limitations. First, while our study aimed to conduct a comprehensive search by selecting PubMed, Web of Science, and IEEE, the exclusion of other databases, such as Embase, Scopus, and Google Scholar, may have led to the omission of some relevant studies. PubMed covers biomedical literature extensively, Web of Science offers broad scientific coverage across multiple fields, and IEEE provides access to technological advancements, particularly in AI. Despite this limitation, we believe our search strategy adequately captured key developments and trends in the application of AI to herpes zoster research. Second, our review may have language bias, as we only included articles published in English. This may have excluded valuable insights from non-English publications, particularly those from regions where herpes zoster research is actively conducted. Third, the data collection process, while rigorous and involving multiple researchers, inherently carries the risk of subjective bias when interpreting study relevance and extracting data. We mitigated this risk through independent evaluation and consensus discussions. However, minor biases may remain. Finally, the rapid evolution of AI technologies, especially the emergence of large models and multimodal learning approaches, presents a challenge for comprehensive coverage. Large language models and multimodal AI systems, which can integrate diverse data types such as clinical notes, imaging, and genomic data, are increasingly being applied in medical research. Some cutting-edge developments might have been missed.

### Conclusions

In summary, this study provides a comprehensive synthesis of the current landscape of AI applications in herpes zoster research, highlighting key trends, methodologies, and challenges. AI has demonstrated significant potential in enhancing the diagnosis, treatment, and management of herpes zoster, with NN-based classification models emerging as the dominant approach. Despite the growing research interest and technological advancements, the findings reveal a lack of international collaborations, indicating the need for greater interdisciplinary and cross-border cooperation. Furthermore, the reliance on hospital-derived data, particularly medical images and EMRs, underscores the importance of data quality and accessibility in AI-driven research. Moving forward, fostering collaborative research efforts, integrating diverse data sources, and exploring novel AI methodologies will be crucial to advancing this field. This study serves as a valuable reference for researchers and clinicians, providing insights that can guide future studies and the development of AI-driven solutions for herpes zoster diagnosis, treatment, and management.

## Supplementary material

10.2196/71970 Multimedia Appendix 1 Search formulas for each databases, and studies about artificial intelligence application in herpes zoster included for analysis in this study.

10.2196/71970 Multimedia Appendix 2 Quality assessment results of included studies using JBI critical appraisal checklist for analytical cross-sectional studies.

10.2196/71970 Checklist 1 PRISMA (Preferred Reporting Items for Systematic Reviews and Meta-Analyses) checklist.

## Abbreviations

AI: artificial intelligence
CNN: convolutional neural network
DNN: deep neural network
EEG: electroencephalogram
EMR: electronic medical record
fMRI: functional magnetic resonance imaging
JBI: Joanna Briggs Institute
k-NN: k-nearest neighbor
ML: machine learning
MRP: medication-resistant pain
MSHA: mixed-scale hierarchical attention
NLP: natural language processing
NN: neural network
PHN: postherpetic neuralgia
PRISMA: Preferred Reporting Items for Systematic Reviews and Meta-Analyses
RF: random forest
SVM: support vector machine
VZV: varicella-zoster virus
