# Supplementary material for: Advancements in Herpes Zoster Diagnosis, Treatment, and Management: Systematic Review of Artificial Intelligence Applications
Source: J Med Internet Res. 2025 Jun 30;27:e71970. doi: 10.2196/71970 (PMC12234400; doi:10.2196/71970)
Supplement: Multimedia Appendix 1 [file jmir-v27-e71970-s001.docx]

**Table S1.** Search formulas and search results from different databases.

| **Search databases** | **Search formulas** | **Search results** | **Total studies** |
| --- | --- | --- | --- |
| PubMed | (("herpes zoster"[Title/Abstract]) OR ("shingles"[Title/Abstract]) OR ("zoster"[Title/Abstract]) OR ("postherpetic neuralgia"[Title/Abstract])) AND (("artificial intelligence"[MeSH Terms]) OR ("machine learning"[MeSH Terms]) OR ("deep learning"[Title/Abstract]) OR ("natural language processing"[Title/Abstract]) OR ("data mining"[Title/Abstract]) OR ("image analysis"[Title/Abstract]) OR ("pattern recognition"[Title/Abstract]) OR ("computer vision"[Title/Abstract])) | 65 | 161 |
| Web of Science Core Collection | TS = (("herpes zoster") OR ("shingles") OR ("zoster") OR ("postherpetic neuralgia")) AND TS = (("artificial intelligence") OR ("machine learning") OR ("deep learning") OR ("natural language processing") OR ("data mining") OR ("image analysis") OR ("pattern recognition") OR ("computer vision")) | 84 |  |
| IEEE | Journals & Magazines: (("herpes zoster") OR ("shingles") OR ("zoster") OR ("postherpetic neuralgia")) AND (("artificial intelligence") OR ("machine learning") OR ("deep learning") OR ("natural language processing") OR ("data mining") OR ("image analysis") OR ("pattern recognition") OR ("computer vision")) | 12 |  |

**Table S2** Main studies about AI application in herpes zoster included for analysis in this study.

| **No** | **Title** | **Organization** | **Publication_Year** | **DOI_Link** | **PubMed_ID** | **WOS_ID** |
| --- | --- | --- | --- | --- | --- | --- |
| 1 | Development of a Novel Multi-Modal Contextual Fusion Model for Early Detection of Varicella Zoster Virus Skin Lesions in Human Subjects | Near East University; Near East University; University of Sharjah; Near East University; University of Sharjah | 2023 | http://dx.doi.org/10.3390/pr11082268 | NA | WOS:001055738500001 |
| 2 | Exploring machine learning methods for predicting systemic lupus erythematosus with herpes | Southwest Medical University; Southwest Medical University; Southwest Medical University; Southwest Medical University | 2023 | http://dx.doi.org/10.1111/1756-185X.14869 | 37578132 | WOS:001048220200001 |
| 3 | In Silico Design and Experimental Validation of Novel Oxazole Derivatives Against Varicella zoster virus | National Academy of Sciences Ukraine; V. P. Kukhar Institute of Bioorganic Chemistry & Petrochemistry of the National Academy of Sciences of Ukraine; University of Alabama System; University of Alabama Birmingham | 2023 | http://dx.doi.org/10.1007/s12033-023-00670-w | 36709460 | WOS:000923349900001 |
| 4 | Characterization of plasma metabolites and proteins in patients with herpetic neuralgia and development of machine learning predictive models based on metabolomic profiling | Sichuan University; Sichuan University; Sichuan University | 2022 | http://dx.doi.org/10.3389/fnmol.2022.1009677 | 36277496 | WOS:000874576700001 |
| 5 | Low-cost image analysis with convolutional neural network for herpes zoster | Pontificia Universidad Catolica del Peru; Universidad Tecnologica del Peru | 2022 | http://dx.doi.org/10.1016/j.bspc.2021.103250 | NA | WOS:000710813800006 |
| 6 | Meibomian gland morphological changes in ocular herpes zoster patients based on AI analysis | Wenzhou Medical University; Zhejiang Chinese Medical University | 2022 | http://dx.doi.org/10.3389/fcell.2022.1094044 | 36531951 | WOS:000898069000001 |
| 7 | EEG Beta-Band Spectral Entropy Can Predict the Effect of Drug Treatment on Pain in Patients With Herpes Zoster | Shenzhen University; Shenzhen University; Guangdong Medical University; Guangdong Medical University; Peng Cheng Laboratory | 2022 | http://dx.doi.org/10.1097/WNP.0000000000000758 | 32675727 | WOS:000796700200010 |
| 8 | A Deep Learning Approach to Estimate the Incidence of Infectious Disease Cases for Routinely Collected Ambulatory Records: The Example of Varicella-Zoster | University of Padua; University of Padua | 2022 | http://dx.doi.org/10.3390/ijerph19105959 | 35627495 | WOS:000803546500001 |
| 9 | Application of Natural Language Processing to Identify Varicella Zoster Infection in Clinical Notes | University of California System; University of California Berkeley; University of California System; University of California San Francisco; University of California System; University of California San Francisco; University of California System; University of California San Francisco; University of California System; University of California San Francisco | 2022 | NA | NA | WOS:000877386501248 |
| 10 | The Curative Effect of Pregabalin in the Treatment of Postherpetic Neuralgia Analyzed by Deep Learning-Based Brain Resting-State Functional Magnetic Resonance Images | | 2022 | http://dx.doi.org/10.1155/2022/2250621 | 35615728 | WOS:000802763600002 |
| 11 | Improved Deep Learning Framework for Segmenting and Classifying Skin Lesions using K-nearest neighbor algorithm and Multi-Instance Learning | Department of Electronics And Communication, T.John Institute Of Technology, Karnataka, India; Department of Electronics and Telecommunication Engineering, St. Francis Institute of Technology; Department of Electrical and Electronics Engineering, BITS Pilani; Department of Mechanical Engineering, Mahatma Gandhi Institute of Technology, Gandipet, India; Department of Computer Engineering Engineering, P.E.S Modern College of Engineering, Pune, India; Department of Information and Communication Technology, DAIICT, Gandhinagar, India | 2022 | NA | NA | NA |
| 12 | Text-Based Identification of Herpes Zoster Ophthalmicus With Ocular Involvement in the Electronic Health Record: A Population-Based Study | Kaiser Permanente; Kaiser Permanente | 2021 | http://dx.doi.org/10.1093/ofid/ofaa652 | NA | WOS:000637323400038 |
| 13 | Robust Skin Disease Classification by Distilling Deep Neural Network Ensemble for the Mobile Diagnosis of Herpes Zoster | Gwangju Institute of Science & Technology (GIST); Yonsei University; Yonsei University Health System | 2021 | http://dx.doi.org/10.1109/ACCESS.2021.3054403 | NA | WOS:000615038100001 |
| 14 | Clinical Characteristics, Treatment Effectiveness, and Predictors of Response to Pharmacotherapeutic Interventions Among Patients with Herpetic-Related Neuralgia: A Retrospective Analysis | Shenzhen University; Chinese University of Hong Kong; Chinese University of Hong Kong | 2021 | http://dx.doi.org/10.1007/s40122-021-00303-7 | 34510386 | WOS:000695102300001 |
| 15 | A convolutional neural network architecture for the recognition of cutaneous manifestations of COVID-19 | All India Institute of Medical Sciences (AIIMS) New Delhi | 2021 | http://dx.doi.org/10.1111/dth.14902 | 33604961 | WOS:000622927700001 |
| 16 | Predicting Postherpetic Neuralgia in Patients with Herpes Zoster by Machine Learning: A Retrospective Study | Beijing University of Chinese Medicine; China-Japan Friendship Hospital | 2020 | http://dx.doi.org/10.1007/s40122-020-00196-y | 32915399 | WOS:000568466200001 |
| 17 | Development, Implementation, and Evaluation of a Personalized Machine Learning Algorithm for Clinical Decision Support: Case Study With Shingles Vaccination | New York University; New York University; New York University | 2020 | http://dx.doi.org/10.2196/16848 | 32347813 | WOS:000529305600001 |
| 18 | Study on the related factors of post-herpetic neuralgia in hospitalized patients with herpes zoster in Sichuan Hospital of Traditional Chinese Medicine based on big data analysis | Chengdu University of Traditional Chinese Medicine; Chengdu University of Traditional Chinese Medicine; Chengdu University of Traditional Chinese Medicine | 2020 | http://dx.doi.org/10.1111/dth.14410 | 33052606 | WOS:000583794200001 |
| 19 | AI-based detection of erythema migrans and disambiguation against other skin lesions | Johns Hopkins University; Johns Hopkins University Applied Physics Laboratory; Johns Hopkins University; Johns Hopkins University | 2020 | http://dx.doi.org/10.1016/j.compbiomed.2020.103977 | 32949845 | WOS:000587354800017 |
| 20 | Using natural language processing for identification of herpes zoster ophthalmicus cases to support population-based study | Kaiser Permanente; Kaiser Permanente; University of California System; University of California Los Angeles; University of California Los Angeles Medical Center; Kaiser Permanente | 2019 | http://dx.doi.org/10.1111/ceo.13340 | 29920898 | WOS:000458614400002 |
| 21 | Automated detection of erythema migrans and other confounding skin lesions via deep learning | Johns Hopkins University; Johns Hopkins University Applied Physics Laboratory; Johns Hopkins University; Johns Hopkins University; Johns Hopkins University | 2019 | http://dx.doi.org/10.1016/j.compbiomed.2018.12.007 | 30654165 | WOS:000458943300015 |
| 22 | Quantifying the incidence and burden of herpes zoster in New Zealand general practice: a retrospective cohort study using a natural language processing software inference algorithm | University of Auckland; University of Otago | 2018 | http://dx.doi.org/10.1136/bmjopen-2017-021241 | 29858420 | WOS:000435567200154 |
| 23 | Statistical Learning Methods to Determine Immune Correlates of Herpes Zoster in Vaccine Efficacy Trials | Fred Hutchinson Cancer Center | 2018 | http://dx.doi.org/10.1093/infdis/jiy421 | 30247601 | WOS:000448167700008 |
| 24 | Social media for arthritis-related comparative effectiveness and safety research and the impact of direct-to-consumer advertising | University of Alabama System; University of Alabama Birmingham | 2017 | http://dx.doi.org/10.1186/s13075-017-1251-y | 28270190 | WOS:000396276000007 |
| 25 | Performance of Machine Learning Methods Using Electronic Medical Records to Predict Varicella Zoster Virus Infection | University of California System; University of California San Francisco; University of California System; University of California San Francisco; US Department of Veterans Affairs; Veterans Health Administration (VHA); San Francisco VA Medical Center; University of California System; University of California San Francisco; Stanford University; Stanford University | 2017 | NA | NA | WOS:000411824106394 |
| 26 | Using Machine Learning for Automatic Identification of Evidence-Based Health Information on the Web | University of Brighton; University of Brighton; University of Sussex | 2017 | http://dx.doi.org/10.1145/3079452.3079470 | NA | WOS:000850447100026 |

**Reference**

1. Eze MC, Vafaei LE, Eze CT, Tursoy T, Ozsahin DU, Mustapha MT. Development of a novel multi-modal contextual fusion model for early detection of varicella zoster virus skin lesions in human subjects. Processes. 2023;11(8):2268.

2. Wang DC, Tang YY, He CS, Fu L, Liu XY, Xu WD. Exploring machine learning methods for predicting systemic lupus erythematosus with herpes. Int J Rheum Dis. 2023 Oct;26(10):2047-54. PMID: 37578132. doi: 10.1111/1756-185X.14869.

3. Kovalishyn V, Severin O, Kachaeva M, Kobzar O, Keith KA, Harden EA, et al. In Silico Design and Experimental Validation of Novel Oxazole Derivatives Against Varicella zoster virus. Mol Biotechnol. 2024 Apr;66(4):707-17. PMID: 36709460. doi: 10.1007/s12033-023-00670-w.

4. Zhou R, Li J, Zhang Y, Xiao H, Zuo Y, Ye L. Characterization of plasma metabolites and proteins in patients with herpetic neuralgia and development of machine learning predictive models based on metabolomic profiling. Front Mol Neurosci. 2022;15:1009677. PMID: 36277496. doi: 10.3389/fnmol.2022.1009677.

5. Lara JVM, Velásquez RMA. Low-cost image analysis with convolutional neural network for herpes zoster. Biomedical Signal Processing and Control. 2022;71:103250.

6. Yu X, Jia X, Zhang Z, Fu Y, Zhai J, Chen N, et al. Meibomian gland morphological changes in ocular herpes zoster patients based on AI analysis. Front Cell Dev Biol. 2022;10:1094044. PMID: 36531951. doi: 10.3389/fcell.2022.1094044.

7. Wei M, Liao Y, Liu J, Li L, Huang G, Huang J, et al. EEG Beta-Band Spectral Entropy Can Predict the Effect of Drug Treatment on Pain in Patients With Herpes Zoster. J Clin Neurophysiol. 2022 Feb 1;39(2):166-73. PMID: 32675727. doi: 10.1097/WNP.0000000000000758.

8. Lanera C, Baldi I, Francavilla A, Barbieri E, Tramontan L, Scamarcia A, et al. A Deep Learning Approach to Estimate the Incidence of Infectious Disease Cases for Routinely Collected Ambulatory Records: The Example of Varicella-Zoster. Int J Environ Res Public Health. 2022 May 13;19(10). PMID: 35627495. doi: 10.3390/ijerph19105959.

9. Ho A, Izadi Z, Schmajuk G, Yazdany J, Tamang S, Gianfrancesco M, editors. Application of Natural Language Processing to Identify Varicella Zoster Infection in Clinical Notes. ARTHRITIS & RHEUMATOLOGY; 2022: WILEY 111 RIVER ST, HOBOKEN 07030-5774, NJ USA.

10. Zheng S, Lei M, Bai F, Tian Z, Wang H. The Curative Effect of Pregabalin in the Treatment of Postherpetic Neuralgia Analyzed by Deep Learning-Based Brain Resting-State Functional Magnetic Resonance Images. Contrast Media Mol Imaging. 2022;2022:2250621. PMID: 35615728. doi: 10.1155/2022/2250621.

11. Nayak A, Shiromani S, Hemanth N, Dekhane A, Patel N, editors. Improved Deep Learning Framework for Segmenting and Classifying Skin Lesions using K-nearest neighbor algorithm and Multi-Instance Learning. 2022 4th International Conference on Advances in Computing, Communication Control and Networking (ICAC3N); 2022: IEEE.

12. Zheng C, Sy LS, Tanenbaum H, Tian Y, Luo Y, Ackerson B, et al., editors. Text-based identification of herpes zoster ophthalmicus with ocular involvement in the electronic health record: a population-based study. Open Forum Infectious Diseases; 2021: Oxford University Press US.

13. Back S, Lee S, Shin S, Yu Y, Yuk T, Jong S, et al. Robust skin disease classification by distilling deep neural network ensemble for the mobile diagnosis of herpes zoster. IEEE Access. 2021;9:20156-69.

14. Zhou J, Sun W, Liu Y, Yang S, Wu S, Wang S, et al. Clinical Characteristics, Treatment Effectiveness, and Predictors of Response to Pharmacotherapeutic Interventions Among Patients with Herpetic-Related Neuralgia: A Retrospective Analysis. Pain Ther. 2021 Dec;10(2):1511-22. PMID: 34510386. doi: 10.1007/s40122-021-00303-7.

15. Mathur J, Chouhan V, Pangti R, Kumar S, Gupta S. A convolutional neural network architecture for the recognition of cutaneous manifestations of COVID-19. Dermatol Ther. 2021 Mar;34(2):e14902. PMID: 33604961. doi: 10.1111/dth.14902.

16. Wang XX, Zhang Y, Fan BF. Predicting Postherpetic Neuralgia in Patients with Herpes Zoster by Machine Learning: A Retrospective Study. Pain Ther. 2020 Dec;9(2):627-35. PMID: 32915399. doi: 10.1007/s40122-020-00196-y.

17. Chen J, Chokshi S, Hegde R, Gonzalez J, Iturrate E, Aphinyanaphongs Y, et al. Development, Implementation, and Evaluation of a Personalized Machine Learning Algorithm for Clinical Decision Support: Case Study With Shingles Vaccination. J Med Internet Res. 2020 Apr 29;22(4):e16848. PMID: 32347813. doi: 10.2196/16848.

18. Li T, Wang J, Xie H, Hao P, Qing C, Zhang Y, et al. Study on the related factors of post-herpetic neuralgia in hospitalized patients with herpes zoster in Sichuan Hospital of Traditional Chinese Medicine based on big data analysis. Dermatol Ther. 2020 Nov;33(6):e14410. PMID: 33052606. doi: 10.1111/dth.14410.

19. Burlina PM, Joshi NJ, Mathew PA, Paul W, Rebman AW, Aucott JN. AI-based detection of erythema migrans and disambiguation against other skin lesions. Comput Biol Med. 2020 Oct;125:103977. PMID: 32949845. doi: 10.1016/j.compbiomed.2020.103977.

20. Zheng C, Luo Y, Mercado C, Sy L, Jacobsen SJ, Ackerson B, et al. Using natural language processing for identification of herpes zoster ophthalmicus cases to support population-based study. Clin Exp Ophthalmol. 2019 Jan;47(1):7-14. PMID: 29920898. doi: 10.1111/ceo.13340.

21. Burlina PM, Joshi NJ, Ng E, Billings SD, Rebman AW, Aucott JN. Automated detection of erythema migrans and other confounding skin lesions via deep learning. Comput Biol Med. 2019 Feb;105:151-6. PMID: 30654165. doi: 10.1016/j.compbiomed.2018.12.007.

22. Turner NM, MacRae J, Nowlan ML, McBain L, Stubbe MH, Dowell A. Quantifying the incidence and burden of herpes zoster in New Zealand general practice: a retrospective cohort study using a natural language processing software inference algorithm. BMJ Open. 2018 May 31;8(5):e021241. PMID: 29858420. doi: 10.1136/bmjopen-2017-021241.

23. Gilbert PB, Luedtke AR. Statistical Learning Methods to Determine Immune Correlates of Herpes Zoster in Vaccine Efficacy Trials. J Infect Dis. 2018 Sep 22;218(suppl_2):S99-S101. PMID: 30247601. doi: 10.1093/infdis/jiy421.

24. Curtis JR, Chen L, Higginbotham P, Nowell WB, Gal-Levy R, Willig J, et al. Social media for arthritis-related comparative effectiveness and safety research and the impact of direct-to-consumer advertising. Arthritis Res Ther. 2017 Mar 7;19(1):48. PMID: 28270190. doi: 10.1186/s13075-017-1251-y.

25. Gianfrancesco M, Schmajuk G, Murray S, Ludwig D, Hannun A, Avati A, et al., editors. Performance of Machine Learning Methods Using Electronic Medical Records to Predict Varicella Zoster Virus Infection. ARTHRITIS & RHEUMATOLOGY; 2017: WILEY 111 RIVER ST, HOBOKEN 07030-5774, NJ USA.

26. Al-Jefri MM, Evans R, Ghezzi P, Uchyigit G, editors. Using machine learning for automatic identification of evidence-based health information on the web. Proceedings of the 2017 international conference on digital health; 2017.
