# Supplementary material for: Advancements in Herpes Zoster Diagnosis, Treatment, and Management: Systematic Review of Artificial Intelligence Applications
Source: J Med Internet Res. 2025 Jun 30;27:e71970. doi: 10.2196/71970 (PMC12234400; doi:10.2196/71970)
Supplement: Multimedia Appendix 2 [file jmir-v27-e71970-s002.docx]

The quality of the included studies was assessed using the Joanna Briggs Institute (JBI) critical appraisal tool. In the assessment table, “Y” stands for “Yes”, “N” for “No”, and “U” for “Unclear”. The quality score for each study was calculated based on the number of “Yes” responses. The quality of the studies was categorized as follows: a score ≥6 was considered high quality, a score ≥4 and <6 was considered moderate quality, and a score <4 was considered low quality. The tool includes the following eight questions: 1) Were the criteria for inclusion in the sample clearly defined? 2) Were the study subjects and the setting described in detail? 3) Was the exposure measured in a valid and reliable way? 4) Were objective, standard criteria used for measurement of the condition? 5) Were confounding factors identified? 6) Were strategies to deal with confounding factors stated? 7) Were the outcomes measured in a valid and reliable way? 8) Was appropriate statistical analysis used?

**Table S3.** Quality assessment results of included studies using JBI critical appraisal checklist for analytical cross-sectional studies.

| Study | JBI’s critical appraisal questions | | | | | | | | Score | Overall Appraisal |
| --- | --- | --- | --- | --- | --- | --- | --- | --- | --- | --- |
|  | Q1 | Q2 | Q3 | Q4 | Q5 | Q6 | Q7 | Q8 |  |  |
| Eze et al [1] . | Y | U | Y | Y | N | N | N | Y | 4 | Medium |
| Wang et al [2] . | Y | Y | Y | Y | Y | Y | Y | Y | 8 | High |
| Kovalishyn et al [3] . | Y | N | Y | Y | N | U | Y | Y | 5 | Medium |
| Zhou et al [4] . | Y | Y | Y | Y | Y | U | Y | Y | 7 | High |
| Mejia et al [5] . | Y | Y | U | U | N | N | U | N | 2 | Low |
| Yu et al [6] . | Y | Y | Y | Y | Y | Y | Y | Y | 8 | High |
| Wei et al [7] . | U | N | Y | Y | U | N | Y | Y | 4 | Medium |
| Lanera et al [8] . | Y | Y | Y | Y | U | N | Y | Y | 6 | High |
| Ho et al [9] . | Y | Y | Y | Y | N | N | Y | Y | 6 | High |
| Zheng et al [10] . | Y | Y | Y | Y | U | U | Y | Y | 6 | High |
| K. S. P et al [11] . | Y | Y | Y | Y | N | N | Y | Y | 6 | High |
| Zheng et al [12] . | Y | Y | Y | Y | Y | Y | Y | Y | 8 | High |
| Back et al [13] . | Y | Y | U | Y | N | N | Y | Y | 5 | Medium |
| Zhou et al [14] . | Y | Y | U | Y | Y | Y | Y | Y | 7 | High |
| Mathur et al [15] . | Y | Y | Y | Y | U | U | Y | Y | 6 | High |
| Wang et al [16] . | Y | Y | Y | Y | Y | Y | Y | Y | 8 | High |
| Chen et al [17] . | Y | Y | Y | Y | Y | Y | Y | Y | 8 | High |
| Li et al [18] . | Y | Y | Y | Y | Y | Y | Y | Y | 8 | High |
| Burlina et al [19] . | Y | Y | Y | Y | Y | Y | Y | Y | 8 | High |
| Zheng et al [20] . | Y | Y | Y | Y | Y | Y | Y | Y | 8 | High |
| Burlina et al [21] . | Y | Y | Y | Y | U | U | Y | Y | 6 | High |
| Turner et al [22] . | Y | Y | Y | Y | Y | Y | Y | Y | 8 | High |
| Gilbert et al [23] . | Y | Y | Y | Y | Y | Y | Y | Y | 8 | High |
| Curtis et al [24] . | Y | Y | Y | Y | U | N | Y | Y | 6 | High |
| Gianfrancesco et al [25] . | Y | Y | Y | Y | U | U | Y | Y | 6 | High |
| Al-Jefri et al [26] . | Y | Y | Y | Y | U | U | Y | Y | 6 | High |

**Reference**

1. Eze MC, Vafaei LE, Eze CT, Tursoy T, Ozsahin DU, Mustapha MT. Development of a novel multi-modal contextual fusion model for early detection of varicella zoster virus skin lesions in human subjects. Processes. 2023;11(8):2268.

2. Wang DC, Tang YY, He CS, Fu L, Liu XY, Xu WD. Exploring machine learning methods for predicting systemic lupus erythematosus with herpes. Int J Rheum Dis. 2023 Oct;26(10):2047-54. PMID: 37578132. doi: 10.1111/1756-185X.14869.

3. Kovalishyn V, Severin O, Kachaeva M, Kobzar O, Keith KA, Harden EA, et al. In Silico Design and Experimental Validation of Novel Oxazole Derivatives Against Varicella zoster virus. Mol Biotechnol. 2024 Apr;66(4):707-17. PMID: 36709460. doi: 10.1007/s12033-023-00670-w.

4. Zhou R, Li J, Zhang Y, Xiao H, Zuo Y, Ye L. Characterization of plasma metabolites and proteins in patients with herpetic neuralgia and development of machine learning predictive models based on metabolomic profiling. Front Mol Neurosci. 2022;15:1009677. PMID: 36277496. doi: 10.3389/fnmol.2022.1009677.

5. Lara JVM, Velásquez RMA. Low-cost image analysis with convolutional neural network for herpes zoster. Biomedical Signal Processing and Control. 2022;71:103250.

6. Yu X, Jia X, Zhang Z, Fu Y, Zhai J, Chen N, et al. Meibomian gland morphological changes in ocular herpes zoster patients based on AI analysis. Front Cell Dev Biol. 2022;10:1094044. PMID: 36531951. doi: 10.3389/fcell.2022.1094044.

7. Wei M, Liao Y, Liu J, Li L, Huang G, Huang J, et al. EEG Beta-Band Spectral Entropy Can Predict the Effect of Drug Treatment on Pain in Patients With Herpes Zoster. J Clin Neurophysiol. 2022 Feb 1;39(2):166-73. PMID: 32675727. doi: 10.1097/WNP.0000000000000758.

8. Lanera C, Baldi I, Francavilla A, Barbieri E, Tramontan L, Scamarcia A, et al. A Deep Learning Approach to Estimate the Incidence of Infectious Disease Cases for Routinely Collected Ambulatory Records: The Example of Varicella-Zoster. Int J Environ Res Public Health. 2022 May 13;19(10). PMID: 35627495. doi: 10.3390/ijerph19105959.

9. Ho A, Izadi Z, Schmajuk G, Yazdany J, Tamang S, Gianfrancesco M, editors. Application of Natural Language Processing to Identify Varicella Zoster Infection in Clinical Notes. ARTHRITIS & RHEUMATOLOGY; 2022: WILEY 111 RIVER ST, HOBOKEN 07030-5774, NJ USA.

10. Zheng S, Lei M, Bai F, Tian Z, Wang H. The Curative Effect of Pregabalin in the Treatment of Postherpetic Neuralgia Analyzed by Deep Learning-Based Brain Resting-State Functional Magnetic Resonance Images. Contrast Media Mol Imaging. 2022;2022:2250621. PMID: 35615728. doi: 10.1155/2022/2250621.

11. Nayak A, Shiromani S, Hemanth N, Dekhane A, Patel N, editors. Improved Deep Learning Framework for Segmenting and Classifying Skin Lesions using K-nearest neighbor algorithm and Multi-Instance Learning. 2022 4th International Conference on Advances in Computing, Communication Control and Networking (ICAC3N); 2022: IEEE.

12. Zheng C, Sy LS, Tanenbaum H, Tian Y, Luo Y, Ackerson B, et al., editors. Text-based identification of herpes zoster ophthalmicus with ocular involvement in the electronic health record: a population-based study. Open Forum Infectious Diseases; 2021: Oxford University Press US.

13. Back S, Lee S, Shin S, Yu Y, Yuk T, Jong S, et al. Robust skin disease classification by distilling deep neural network ensemble for the mobile diagnosis of herpes zoster. IEEE Access. 2021;9:20156-69.

14. Zhou J, Sun W, Liu Y, Yang S, Wu S, Wang S, et al. Clinical Characteristics, Treatment Effectiveness, and Predictors of Response to Pharmacotherapeutic Interventions Among Patients with Herpetic-Related Neuralgia: A Retrospective Analysis. Pain Ther. 2021 Dec;10(2):1511-22. PMID: 34510386. doi: 10.1007/s40122-021-00303-7.

15. Mathur J, Chouhan V, Pangti R, Kumar S, Gupta S. A convolutional neural network architecture for the recognition of cutaneous manifestations of COVID-19. Dermatol Ther. 2021 Mar;34(2):e14902. PMID: 33604961. doi: 10.1111/dth.14902.

16. Wang XX, Zhang Y, Fan BF. Predicting Postherpetic Neuralgia in Patients with Herpes Zoster by Machine Learning: A Retrospective Study. Pain Ther. 2020 Dec;9(2):627-35. PMID: 32915399. doi: 10.1007/s40122-020-00196-y.

17. Chen J, Chokshi S, Hegde R, Gonzalez J, Iturrate E, Aphinyanaphongs Y, et al. Development, Implementation, and Evaluation of a Personalized Machine Learning Algorithm for Clinical Decision Support: Case Study With Shingles Vaccination. J Med Internet Res. 2020 Apr 29;22(4):e16848. PMID: 32347813. doi: 10.2196/16848.

18. Li T, Wang J, Xie H, Hao P, Qing C, Zhang Y, et al. Study on the related factors of post-herpetic neuralgia in hospitalized patients with herpes zoster in Sichuan Hospital of Traditional Chinese Medicine based on big data analysis. Dermatol Ther. 2020 Nov;33(6):e14410. PMID: 33052606. doi: 10.1111/dth.14410.

19. Burlina PM, Joshi NJ, Mathew PA, Paul W, Rebman AW, Aucott JN. AI-based detection of erythema migrans and disambiguation against other skin lesions. Comput Biol Med. 2020 Oct;125:103977. PMID: 32949845. doi: 10.1016/j.compbiomed.2020.103977.

20. Zheng C, Luo Y, Mercado C, Sy L, Jacobsen SJ, Ackerson B, et al. Using natural language processing for identification of herpes zoster ophthalmicus cases to support population-based study. Clin Exp Ophthalmol. 2019 Jan;47(1):7-14. PMID: 29920898. doi: 10.1111/ceo.13340.

21. Burlina PM, Joshi NJ, Ng E, Billings SD, Rebman AW, Aucott JN. Automated detection of erythema migrans and other confounding skin lesions via deep learning. Comput Biol Med. 2019 Feb;105:151-6. PMID: 30654165. doi: 10.1016/j.compbiomed.2018.12.007.

22. Turner NM, MacRae J, Nowlan ML, McBain L, Stubbe MH, Dowell A. Quantifying the incidence and burden of herpes zoster in New Zealand general practice: a retrospective cohort study using a natural language processing software inference algorithm. BMJ Open. 2018 May 31;8(5):e021241. PMID: 29858420. doi: 10.1136/bmjopen-2017-021241.

23. Gilbert PB, Luedtke AR. Statistical Learning Methods to Determine Immune Correlates of Herpes Zoster in Vaccine Efficacy Trials. J Infect Dis. 2018 Sep 22;218(suppl_2):S99-S101. PMID: 30247601. doi: 10.1093/infdis/jiy421.

24. Curtis JR, Chen L, Higginbotham P, Nowell WB, Gal-Levy R, Willig J, et al. Social media for arthritis-related comparative effectiveness and safety research and the impact of direct-to-consumer advertising. Arthritis Res Ther. 2017 Mar 7;19(1):48. PMID: 28270190. doi: 10.1186/s13075-017-1251-y.

25. Gianfrancesco M, Schmajuk G, Murray S, Ludwig D, Hannun A, Avati A, et al., editors. Performance of Machine Learning Methods Using Electronic Medical Records to Predict Varicella Zoster Virus Infection. ARTHRITIS & RHEUMATOLOGY; 2017: WILEY 111 RIVER ST, HOBOKEN 07030-5774, NJ USA.

26. Al-Jefri MM, Evans R, Ghezzi P, Uchyigit G, editors. Using machine learning for automatic identification of evidence-based health information on the web. Proceedings of the 2017 international conference on digital health; 2017.
